# Validation of the Internet Addiction Test (IAT) for forest firefighters: implications for human–technology interaction and occupational safety in the future of work

**DOI:** 10.3389/fpsyg.2026.1827523

**Published:** 2026-06-19

**Authors:** Carla Estrada-Muñoz, Felipe Meyer-Cohen, Mauricio E. Garrido Vásquez, Jean Paul Navarrete-Campos, Alejandro Vega-Muñoz, Guido Salazar-Sepúlveda

**Affiliations:** 1 Departamento de Ergonomía, Facultad de Ciencias Biológicas, Universidad de Concepción, Concepción, Chile; 2 Departamento de Psicología, Facultad de Ciencias Sociales, Universidad de Concepción, Concepción, Chile; 3 Departamento de Estadística, Facultad de Ciencias Físicas y Matemáticas, Universidad de Concepción, Concepción, Chile; 4 Laboratorio de Bienestar y Comportamiento Organizacional, Facultad de Medicina y Ciencias de la Salud, Universidad Central de Chile, Santiago, Chile; 5 Centro de Investigación en Medicina de Altura, Universidad Arturo Prat, Santiago, Chile; 6 Facultad de Ingeniería, Universidad Católica de la Santísima Concepción, Concepción, Chile; 7 Facultad de Ingeniería y Negocios, Universidad de Las Américas, Concepción, Chile

**Keywords:** attentional functioning, cognitive load, digital fatigue, digital job demands, digital psychosocial risks, occupational wellbeing, organizational behavior, technological self-regulation

## Abstract

The digitization of emergency response work has intensified human–technology interaction and altered the cognitive and organizational demands placed on wildland firefighters. In this study, we validated the Internet Addiction Test (IAT) in a sample of 205 wildland firefighters to identify patterns of digital use that may compromise attention, sleep, and operational safety. Factor and internal consistency analyses supported a two-factor structure that distinguished between a broad pattern of problematic engagement and more specific behaviors of loss of control; the confirmatory model showed excellent fit (CFI = 0.997; TLI = 0.997; RMSEA = 0.026; SRMR = 0.063). Although discriminant validity was limited by high inter-factor correlation, the scale exhibits adequate reliability for use as an occupational screening tool. We propose that the IAT be used in combination with ergonomic indicators and telemetry (e.g., device usage logs, fatigue and performance measures) to distinguish necessary operational use from maladaptive use and to guide interventions in digital governance, alert design, and training in technological self-regulation. These results provide a context-sensitive measure for managing digital risks in emergency services and guide future longitudinal research linking IAT scores to safety and performance outcomes.

## Introduction

The rapid expansion of information and communication technologies (ICT) has fundamentally reshaped how information is produced, managed, and transmitted, transforming social, organizational, and work environments worldwide (Castells, 2010; UNESCO, 2009). Digital platforms, mobile devices, pervasive connectivity, and data-driven systems now form the backbone of contemporary professional activity, accelerating human–technology interaction and redefining the competencies required for the future of work (Laudon and Laudon, 2020; Autor et al., 2020). In this context, emerging debates on new professionalism emphasize how digitalization is altering professional identities, cognitive demands, and expectations of performance across sectors, particularly in occupations where technology mediates critical decisions and real-time coordination. Although digitalization enables automation, operational efficiency, and temporal and spatial flexibility, it also creates persistent challenges—unequal access, rapid obsolescence, information-management risks, and growing dependence on digital tools for routine tasks—that extend beyond purely technical concerns (Eurofound, 2020; Park, 2026; Lam and Harcourt, 2024).

As workplaces become more digitally mediated, the cognitive and emotional demands placed on workers intensify. Scholars have documented a range of adverse outcomes associated with pervasive ICT use (technostress, information overload, sleep disruption, and impaired decision processes) that undermine wellbeing and performance (Nastjuk et al., 2024; Phillips et al., 2024; Blake et al., 2024). Problematic or compulsive patterns of technology use (often termed technoaddiction or problematic smartphone/social media use) are associated with anxiety, depression, attentional difficulties, and reduced occupational functioning (Griffiths, 2005; Carbonell et al., 2018; Gao and Shao, 2024). These phenomena are shaped by individual predispositions (e.g., impulsivity, poor self-regulation), organizational practices (e.g., expectations of constant availability), and broader sociocultural pressures toward permanent connectivity (Pontes, 2017; Mouakket and Aboelmaged, 2023; Tarafdar et al., 2019). From a work-psychology perspective, these dynamics position digital job demands and technology-driven pressures as central psychosocial risks in modern organizations.

The manifestations of technoaddiction encompass various behaviors linked to problematic use of the Internet, social media, and video games, which have been widely documented in specialized literature (Young, 1998; Kuss and Griffiths, 2011). Empirical evidence indicates that its development is due to a combination of factors (Vanea, 2011). At the individual level, variables such as impulsivity, social anxiety, low self-esteem, and certain personality traits have been identified. At the family level, poor communication and the absence of clear boundaries; and in the sociocultural context, the normalization of permanent connectivity and the pressure to maintain a constant digital presence (Pontes, 2017; Muñoz-Rivas et al., 2010; Elhai et al., 2019). In safety-critical and rapidly digitalizing workplaces, problematic technology use must also be understood as a function of work design, organizational norms, and technology-mediated task structures.

Current theoretical frameworks agree that digital technology can be functional when it bridges real gaps in access and training but becomes dysfunctional when it interacts with personal vulnerabilities and designs that reinforce immediate gratification. Cognitive-behavioral models highlight dysfunctional beliefs and reinforcement contingencies (Davis, 2001); the I-PACE model explains how the interaction among personal, affective, cognitive, and executive factors facilitate the transition toward addictive uses (Brand et al., 2019, 2016); and research on persuasive design shows that reward mechanisms built into platforms promote habit formation and loss of control (Alter, 2017; Montag et al., 2021). These perspectives converge on the idea that problematic digital engagement is not merely an individual deficit but an emergent property of person–technology–context interactions.

Problematic digital engagement, understood beyond screen time, reflects deficits in self-regulation and attention that are associated with impaired sleep, mood, and performance. Meta-analytic evidence link is low self-regulation to problematic forms of use (Howard et al., 2025) and studies of technoference show that technological interruptions mediate negative effects on socio-emotional adjustment (Hu, 2025). Moreover, implicit behavioral indices add predictive value for risky behaviors—outperforming self-reports in detecting context-dependent and state-specific risk patterns (Cimino and Cerniglia, 2025). At the functional end of the spectrum, interventions that provide devices, connectivity, and training increase community participation and trust (Drazich et al., 2021; Li et al., 2022). At the dysfunctional end, emotional attachment to devices, overexposure to social media, and altered reward processing convert stress into problematic use and worsen sleep among adolescents (Bata et al., 2018; Servidio et al., 2022; Qi et al., 2025; Pagano et al., 2023). Thus, the same technologies can enable inclusion and capacity building or, under different personal and design conditions, foster maladaptive patterns.

Recent large-scale studies integrating micro-level traits (for example, intolerance of uncertainty, low mindfulness) with contextual stressors (e.g., family conflict, school disengagement) confirm that risks accumulate and interact as predicted by I-PACE (Huang et al., 2026; Cheng et al., 2025). Collective stressors such as the COVID-19 pandemic illustrate how environmental anxiety can transform initially adaptive uses into dysfunctional coping strategies (Elhai et al., 2020). Taken together, these findings imply that interventions should target three linked domains: affective regulation, executive control, and contextual incentives—rather than focusing solely on individual willpower or device removal (Brand et al., 2016, 2019; Huang et al., 2026).

Accurate identification of problematic technology use in occupational settings requires psychometrically robust instruments sensitive to profession-specific demands and risk profiles. The Internet Addiction Test (IAT; Young, 1998) is widely used and shows acceptable reliability and validity across diverse samples (Widyanto and McMurran, 2004; Pontes et al., 2014; Moon et al., 2018). However, applying general-population instruments to safety-critical, field-based responder populations demands re-examination of measurement properties, construct relevance, and criterion associations, because patterns of “essential” vs. “non-essential” Internet use differ markedly from office or student samples (Weidinger, 2022; Fischer-Preßler et al., 2024).

Firefighting—and in particular forest and wildland firefighting—constitutes a paradigmatic safety-critical occupation in which the interplay between technology use and human performance is both intense and consequential. Modern firefighting increasingly relies on internet connectivity via mobile devices, geolocation systems, real-time information feeds, and emergency response information systems to support situational awareness, coordination, and evacuation planning (Weidinger et al., 2023; Weidinger, 2022; Zikeloglou et al., 2024; Fischer-Preßler et al., 2024). At the same time, firefighters face extreme physical, cognitive, and emotional demands, exposure to heat and sleep disruption, and organizational pressures that can amplify vulnerability to maladaptive coping strategies, including problematic digital engagement (Martínez-Fiestas et al., 2020; Paterson et al., 2022; Billings et al., 2024; Lingard et al., 2024). These conditions make firefighters an especially informative case for studying how digitalization reshapes professional roles and introduces new psychosocial risks.

Given the safety implications of impaired attention, delayed decision-making, or distraction in emergency contexts, understanding technology-related vulnerabilities among firefighters is a pressing occupational health priority (Kambarian et al., 2025; García-Iglesias et al., 2025). Empirical work on firefighter acceptance and use of emergency information systems highlights both the potential benefits of digital tools for operational effectiveness and the risks associated with overload, usability limitations, and informal practices that circumvent formal safety procedures (Weidinger et al., 2023; Paterson et al., 2022). Studies of evacuation planning and community response to wildfires emphasize the centrality of reliable, well-designed information flows and the human factors that determine whether digital alerts translate into safe behavior (Zikeloglou et al., 2024). Consequently, measurement tools must distinguish functional, mission-critical technology use from maladaptive patterns that may compromise safety and performance.

Resident firefighters operate in a high-stakes, digitally mediated environment where routine tasks (navigation, incident reporting, and remote coordination) increasingly depend on continuous Internet access and mobile apps; this creates a specific risk that excessive or poorly regulated technology use will impair attention, sleep, mood, and operational performance at critical moments (Mekonnen et al., 2024; Grover et al., 2019). Workplace research documents links between problematic Internet use (PIU) and depression, sleep disturbance, work stress, and functional impairment across diverse occupational groups—medical residents, frontline healthcare workers, airline pilots, teachers, and IT engineers—yet prevalence estimates and measurement approaches vary widely, revealing a theoretical and methodological gap: although the IAT is widely used, its psychometric properties have not been systematically validated for safety-critical, field-based responder populations whose patterns of essential vs. non-essential Internet use differ from office or student samples (Abdul-Hussein et al., 2023; Grover et al., 2019; Aydin et al., 2024; Sun et al., 2024; Fukuda et al., 2023; Chen et al., 2014). For example, recent institutional studies report high PIU prevalence among resident physicians with strong associations to depression and sleep loss, underscoring the need for occupation-specific validation (Mekonnen et al., 2024).

To what extent does the IAT reliably and validly measure problematic technology engagement in forest firefighters, distinguishing operationally necessary connectivity from maladaptive use that threatens safety and wellbeing? Objective: this study validates the Internet Addiction Test (IAT) in a sample of forest firefighters, evaluating its factor structure, internal consistency, and convergent validity with measures of sleep, mood, and work functioning to produce an occupation-specific screening tool for problematic Internet engagement. By situating this validation within the broader transformation of work and the emergence of new forms of professionalism, the study contributes to understanding how digitalization reshapes cognitive demands, professional boundaries, and occupational safety. Ultimately, the research bridges measurement work on technoaddiction with applied concerns about organizational governance, human–technology interaction, operational performance, and the design of healthier digital work environments (Lam and Harcourt, 2024; Obasi and Benson, 2025; Fischer-Preßler et al., 2024).

## Materials and methods

Missing data were handled using multivariate imputation with two complementary approaches. The primary strategy was Multiple Imputation (MI) implemented via Multivariate Imputation by Chained Equations (MICE) using Predictive Mean Matching (PMM). PMM imputes each item from a predictor set formed by the remaining items, preserves the multivariate structure of the scale, and tends to avoid imputations outside the observed support—features that are especially useful for discrete/ordinal variables (van Buuren and Groothuis-Oudshoorn, 2011; Graham, 2009). MI was run with five imputed datasets and 10 iterations per chain; convergence of the chains was inspected. After imputation, values were rounded and constrained to the original 1–5 ordinal range. As a robustness check, k-nearest neighbors' imputation (k-NN; *k* = 5) re-imputed each case from profiles of similar respondents, allowing the stability of the results to be contrasted against an alternative non-parametric method (Troyanskaya et al., 2001). This dual-strategy approach is consistent with best practices in occupational psychology, where robust handling of missing data is essential for ensuring valid inferences in applied, safety-critical populations.

Sample adequacy and assumptions for factor analysis were evaluated prior to modeling. In line with long-standing practical rules of thumb and classical psychometric guidance (Gorsuch, 1983; Nunnally and Bernstein, 1994; Comrey and Lee, 1992), ratios close to 10 participants per item (e.g., *N* > 200 for 20 items) are often considered adequate for simple structures with a small number of factors and moderate-to-high loadings. However, empirical evidence indicates that sample-size requirements depend on the magnitude of communalities and the degree of model overdetermination and can be less stringent when these conditions are favorable (MacCallum et al., 1999). Accordingly, the Kaiser–Meyer–Olkin measure of sampling adequacy (KMO) and Bartlett's test of sphericity were computed as indicators of factorability (Kaiser and Rice, 1974; Bartlett, 1950). Given the applied nature of this study, these diagnostics also ensure that the instrument performs adequately under real-world conditions characteristic of technology-intensive work.

In this study, the Internet Addiction Test (IAT) was used as an assessment tool for maladaptive digital engagement in the occupational context. The purpose of the IAT here is to serve as a screening tool and psychometric measure to identify patterns of use that may be associated with a risk of impairment in sleep, mood, and performance, not as a clinical diagnostic procedure. Therefore, the IAT results should be interpreted as risk indicators and not as diagnostic criteria for a disorder; any case identified as having a high score would require further clinical evaluation. Furthermore, it is acknowledged that the IAT was developed for general populations; thus, in this study, its use is accompanied by analyses of convergent validity and contextual fit to assess its applicability in a population of wildland firefighters.

Following imputation, dimensionality was explored with Exploratory Factor Analysis (EFA) and then tested with a two-factor Confirmatory Factor Analysis (CFA). Given the ordinal response format, CFA was estimated to use methods appropriate for categorical/ordinal indicators (e.g., robust DWLS/WLSMV), which are recommended to improve parameter recovery relative to continuous-variable assumptions for Likert-type items (Rhemtulla et al., 2012; Li, 2016). Model fit was assessed using standard indices—Comparative Fit Index (CFI), Tucker–Lewis Index (TLI), Root Mean Square Error of Approximation (RMSEA), and the Standardized Root Mean Square Residual (SRMR)—drawing on widely used reference criteria and evidence specific to ordinal factor models (Hu and Bentler, 1999; Shi et al., 2020). Internal consistency was evaluated with Cronbach's alpha (α) and McDonald's omega (ω), with ω included as a more general reliability estimator under congeneric measurement (Zinbarg et al., 2005). Convergent validity was quantified via Average Variance Extracted (AVE) and Composite Reliability (CR), and discriminant validity was examined using the Fornell–Larcker criterion (comparing each AVE to the squared inter-factor correlation, r^2^) (Fornell and Larcker, 1981; Raykov, 1997). In addition, the heterotrait–monotrait ratio (HTMT) may be reported as a complementary, contemporary check of discriminant validity (Henseler et al., 2015; Rönkkö and Cho, 2022). This analytic strategy reflects current expectations in work and organizational psychology for validating instruments that assess digital-behavioral risks in evolving work environments.

To strengthen ecological validity, all analyses were conducted with explicit consideration of the occupational context of forest firefighters, a group operating in technology-intensive, high-demand environments where attentional functioning and digital self-regulation are critical for safe performance. This contextual anchoring is consistent with contemporary approaches to human–technology interaction and the study of digital psychosocial risks in the future of work.

Given the ordinal nature of the Likert-type items, polychoric correlations were computed to estimate inter-item associations. The number of factors was determined using parallel analysis (Horn, 1965), and factor extraction was performed using the minimum residual (minres) method with oblimin rotation to allow for correlated factors. Factor loadings ≥ 0.30 were considered meaningful, with values ≥ 0.40 and ≥ 0.60 interpreted as moderate and strong, respectively. Internal consistency was evaluated using Cronbach's alpha (≥0.70; see Table 1).

**Table 1:** Criteria used for psychometric evaluation.

Criterion	Cutoff
KMO	≥0.90 (excellent)
Bartlett's test	*p* < 0.05
Minimum factor loading retained	≥0.30
Cronbach's alpha—Factor 1	≥0.70 (acceptable)
Cronbach's alpha—Factor 2	≥0.70 (acceptable)
Ordinal alpha—Factor 1	≥0.70 (acceptable)
Ordinal alpha—Factor 2	≥0.70 (acceptable)
McDonald's omega—Factor 1	≥0.70 (acceptable)
McDonald's omega—Factor 2	≥0.70 (acceptable)
AVE—Factor 1	≥0.50 (desirable)
AVE—Factor 2	≥0.50 (desirable)
Squared inter-factor correlation	AVE > *r*^2^
CFI	≥0.95 (excellent)
TLI	≥0.95 (excellent)
RMSEA	≤ 0.06 (good fit)
SRMR	≤ 0.08 (acceptable fit)
Missing data	<5% (low)

The proportion of missing data was minimal (0.122% overall), with no item exceeding 0.488%. PMM was used as the primary imputation method to preserve the distribution of ordinal responses, while k-NN imputation and complete-case analyses were conducted as robustness checks. Sensitivity analyses showed consistent results across all approaches, supporting a stable two-factor solution.

All analyses were conducted using R (R Core Team, 2025), with key analyses performed using the *psych, lavaan*, and *mice* packages (Revelle, 2024; Rosseel, 2012). Data preprocessing and manipulation were performed using the packages *dplyr, tidyr*, and *stringr*, while data visualization was carried out using *ggplot2*. Psychometric analyses were conducted using the *psych* package for exploratory factor analysis (EFA) and polychoric correlations, *lavaan* for confirmatory factor analysis (CFA), and *semPlot* for graphical representation of the structural models. Missing data were handled using the *mice* package for multiple imputation via Predictive Mean Matching (PMM), and *VIM* for k-nearest neighbors (k-NN) imputation.

Tables 2, 3 summarize the sociodemographic characteristics of the sample. The study included 205 forest firefighters, predominantly male (92.7%). The mean age was 31.1 years (*SD* = 11.2) and the average number of children was 0.70 (*SD* = 1.05). Most participants were single (73.7%) and had completed secondary education (55.6%), reflecting a relatively young and homogeneous workforce.

**Table 2:** Sociodemographic characteristics of the sample.

Variable	Category	*n*	%
Gender	Male	190	92.7
	Female	15	7.3
Marital status	Single	151	73.7
	Married	31	15.1
	Cohabiting	21	10.2
	Divorced	1	0.5
	Widowed	1	0.5
Education level	Complete secondary	114	55.6
	Incomplete secondary	31	15.1
	Complete primary	25	12.2
	Incomplete primary	14	6.8
	Complete higher education	15	7.3
	Incomplete higher education	6	2.9

**Table 3:** Sociodemographic characteristics of the sample (continuous variables).

Variable	Mean ±SD
Age	31.1 ± 11.2
Children	0.70 ± 1.05

## Results

### Treatment of missing data

Given the ordinal nature of the items on the Likert scale, multiple imputations using the predictive mean matching (PMM) method was selected as the primary method, as it preserves the observed distribution of responses and is better able to preserve the marginal distribution and associations between items. All variables were checked to ensure they were free of “NA” values and that the proportions per category were plausible (i.e., without atypical concentrations at the extremes). A total of 205 responses were obtained. This robust imputation strategy ensures that the psychometric evaluation reflects the actual response patterns of workers operating in high-demand, technology-intensive environments, where missing data may be influenced by workload and cognitive effort. In addition, k-nearest neighbors (k-NN) imputation was performed as a sensitivity analysis to assess the robustness of the results with respect to the imputation method. Complete case analyses were also conducted for comparative purposes. The proportion of missing data was minimal (0.122% in total), with no item exceeding 0.488% missing responses.

In addition, the sensitivity analysis conducted to evaluate the impact of missing data handling on the factorial structure, where compared complete-case data (*N* = 201), PMM-imputed data (*N* = 205), and k-NN-imputed data (*N* = 205), it found that, crossing all datasets, parallel analysis consistently supported a two-factor solution, indicating that the results are robust.

### Sample adequacy and assumptions for factor analysis

The overall KMO value was 0.92; by item, all > 0.82. This indicates excellent sample adequacy: partial correlations are low and the latent pattern is “factorizable.” With the polychoric matrix used and *n* > 200, Bartlett's test was highly significant *p* < 0.001 (rejection of sphericity), comfortably rejecting the sphericity hypothesis. This result is fully consistent with the KMO (0.92) and with the identified factor structure. These diagnostics confirm that the dataset is suitable for modeling latent constructs relevant to digital-behavioral risks in safety-critical occupations.

### Factorial analysis

**Figure 1:**
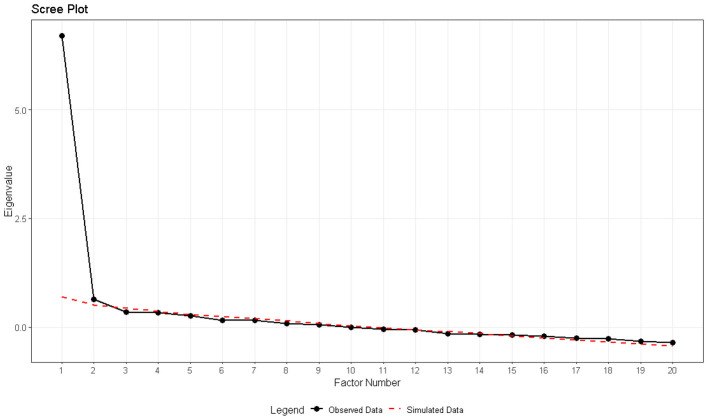
Scree plot with parallel analysis (Horn, 1965).

Figure 1 observed eigenvalues (black solid line) are compared with eigenvalues obtained from randomly generated data (red dashed line). Following the parallel analysis criterion (Horn, 1965), factors are retained when observed eigenvalues exceed their simulated counterparts. The results support a two-factor solution, as only the first two eigenvalues exceed the random-data threshold. The first factor captures a substantial proportion of the shared variance, whereas the second factor contributes additional, more specific variance. Subsequent factors fall below the simulated values, indicating that they likely represent random variation rather than meaningful latent structure.

**Figure 2:**
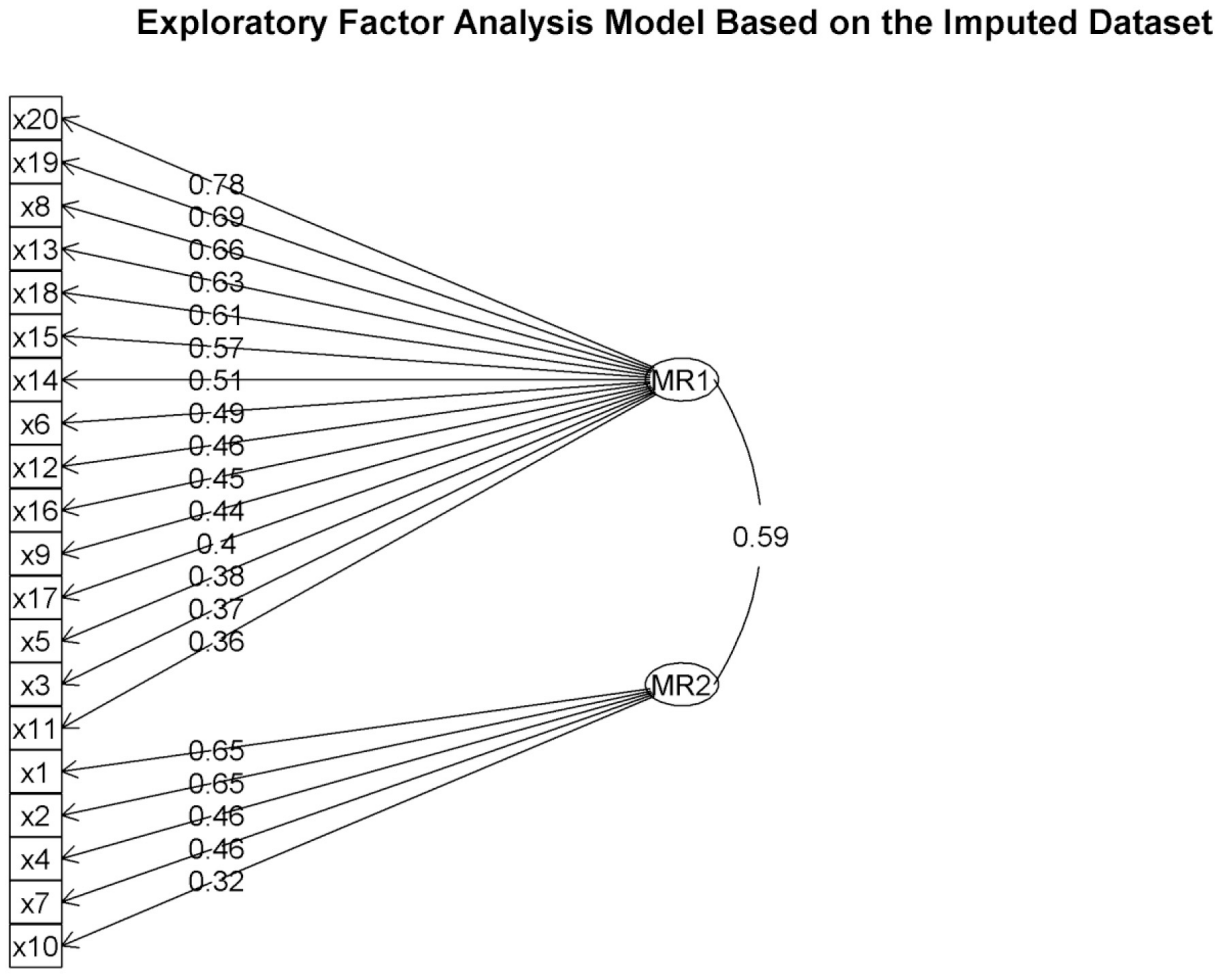
Load diagram by factor (exploratory solution, “MR1” and “MR2”).

Figure 2 shows the latent two-factor structure. The arrows from each factor to the items are the factor loadings (how much the factor explains the item). The correlation between factors in the exploration phase is moderate (~0.59). It can be seen that x3–x20 load on MR1, while x1, x2, x4, and x7 load on MR2. This is the basic conceptual separation of the instrument.

**Figure 3:**
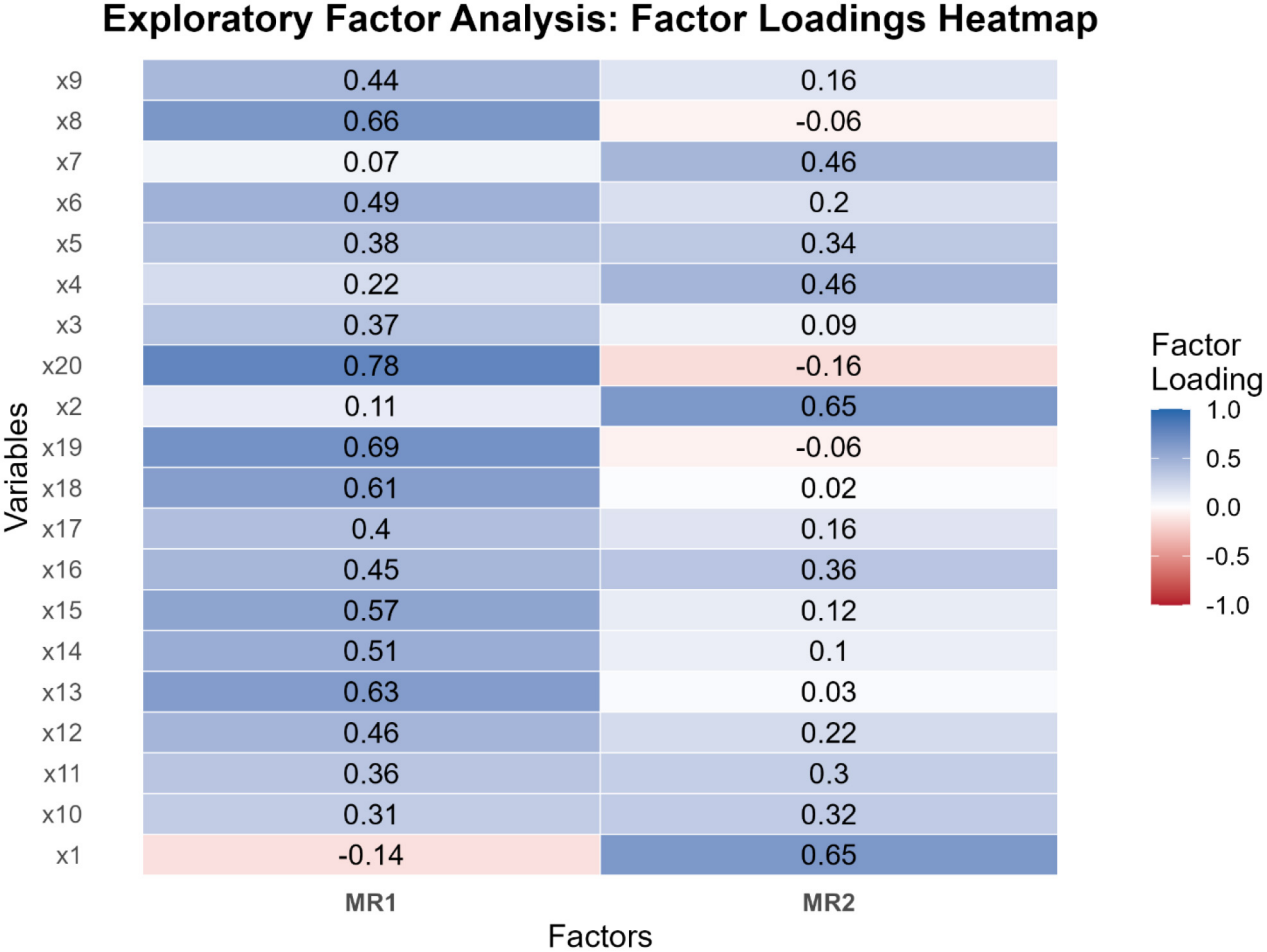
Heat map of factorial loads.

Figure 3 visually confirms the partition: column MR1 shows loads of 0.31–0.78 for x3–x20 (more intense blues), while MR2 concentrates loads of 0.46–0.65 on x1, x2, x4, and x7. The slightly reddish tone of x1 in MR1 reflects a small negative loading (consistent with that item belonging to the other factor).

**Figure 4:**
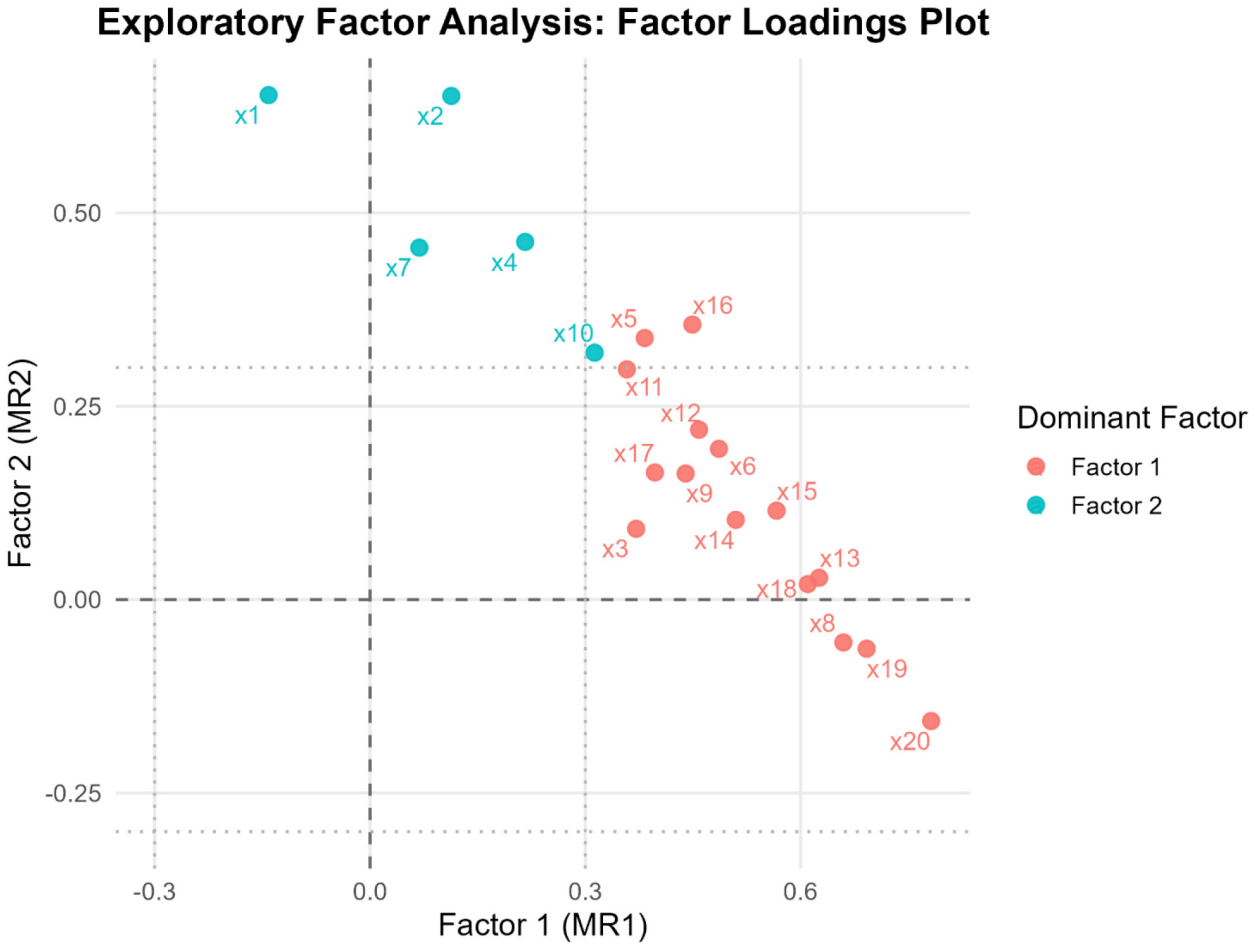
MR1 vs. MR2 factorial plot.

Figure 4 shows that each point is an item located by its loadings on axis 1 (MR1) and axis 2 (MR2). We see a compact group on the right half-axis and close to the horizontal axis (x13, x18, x8, x19, x20...), typical of items that define the first factor; and another group on the upper half-axis (x1, x2, x4, and x7), which defines the second factor. The separation between clouds supports two-dimensionality. This structure suggests that firefighters' technology-related behaviors differentiate between a broad pattern of problematic engagement (F1) and a more specific set of behaviors linked to loss of control or interference with daily functioning (F2), both highly relevant for understanding attentional and self-regulatory demands in emergency work.

**Figure 5:**
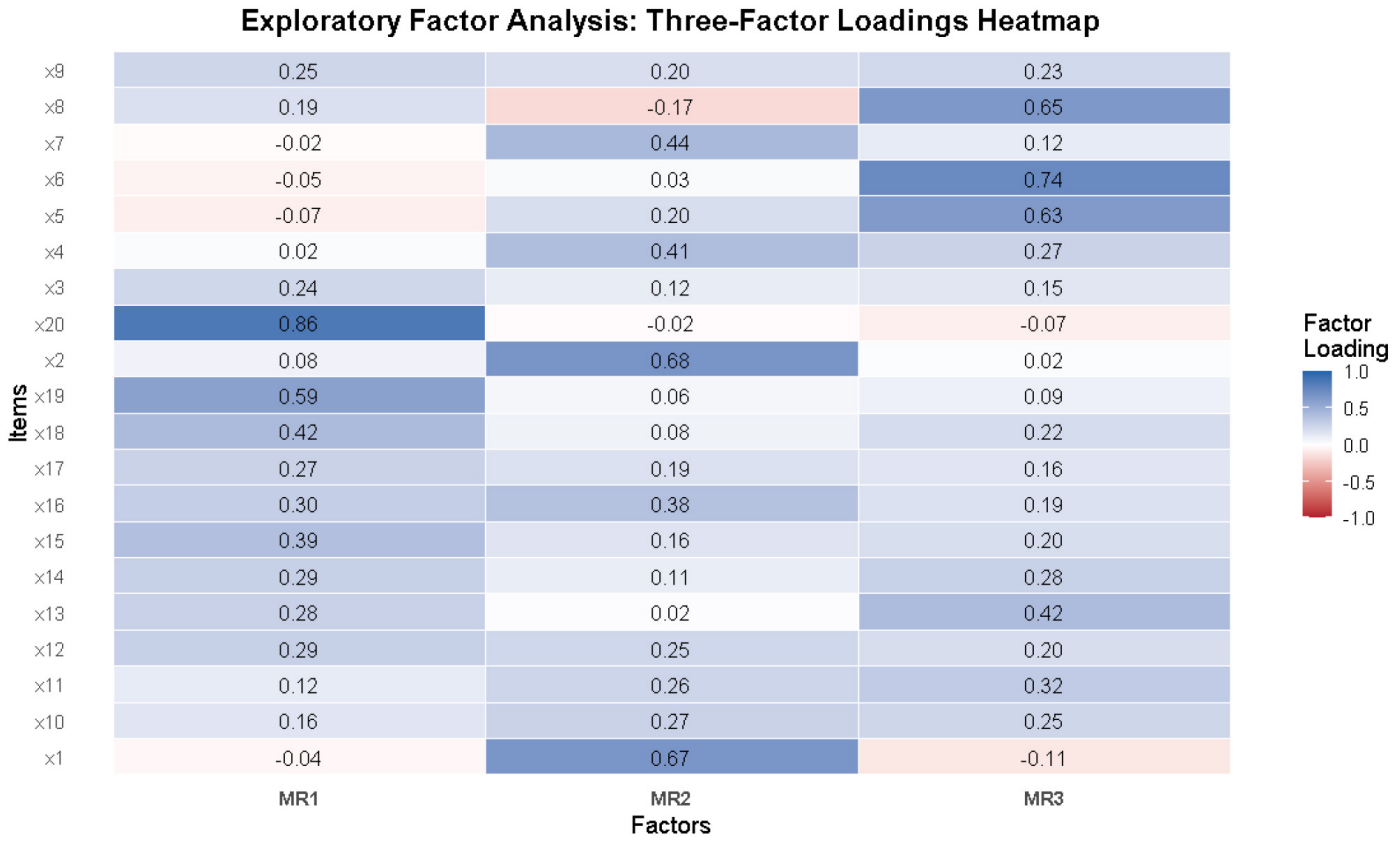
Heatmap of factor loadings for the three-factor solution.

By way of contrast, Figure 5 visually illustrates the distribution of factor loadings across the three-factor solution. While MR1 and MR3 show clusters of moderate to strong loadings (e.g., x20, x19, x6, x5, and x8), MR2 captures a smaller subset of items (notably x1 and x2). However, several items exhibit cross-loadings or relatively low coefficients (e.g., x11, x15, and x16), resulting in a more diffuse and less interpretable structure. This pattern suggests a fragmentation of the underlying construct and is consistent with potential overextraction, reinforcing the preference for the more parsimonious two-factor solution.

Overall, the three-factor solution leads to a fragmentation of the original structure and does not provide a substantively meaningful improvement. This pattern is consistent with potential overextraction, reinforcing the two-factor model as the most parsimonious and theoretically coherent representation of the data.

### Model confirmation (CFA) and fit quality

To evaluate the adequacy of the proposed latent structure and the overall quality of model fit, we compared the obtained psychometric indicators against established benchmarks, as summarized in Table 4.

**Table 4:** Criteria used for psychometric evaluation and obtained results.

Criterion	Cutoff	Obtained value
KMO	≥0.90 (excellent)	0.921
Bartlett's test	*p* < 0.05	*p* < 0.001
Minimum factor loading retained	≥0.30	0.30
Cronbach's alpha—Factor 1	≥0.70 (acceptable)	0.902
Cronbach's alpha—Factor 2	≥0.70 (acceptable)	0.703
Ordinal alpha—Factor 1	≥0.70 (acceptable)	0.930
Ordinal alpha—Factor 2	≥0.70 (acceptable)	0.757
McDonald's omega—Factor 1	≥0.70 (acceptable)	0.905
McDonald's omega—Factor 2	≥0.70 (acceptable)	0.693
AVE—Factor 1	≥0.50 (desirable)	0.467
AVE—Factor 2	≥0.50 (desirable)	0.451
Squared inter-factor correlation	AVE > *r*^2^	0.611
CFI	≥0.95 (excellent)	0.997
TLI	≥0.95 (excellent)	0.997
RMSEA	≤ 0.06 (good fit)	0.026
SRMR	≤ 0.08 (acceptable fit)	0.063
Missing data	<5% (low)	0.122%

The results of the confirmatory factor analysis (CFA) of two correlated factors with ordinal indicators (threshold per category) demonstrated excellent model fit: CFI = 0.997, TLI = 0.997 (both above the excellence threshold of ≥0.95), RMSEA = 0.026 (90% CI: 0.000–0.042), well-below the 0.06 criterion, and SRMR = 0.063, within the acceptable range (<0.08). Prior to this analysis, the KMO index yielded an excellent value (0.921) and Bartlett's test of sphericity was statistically significant (*p* < 0.001), confirming the adequacy of the data for factor analysis. Regarding reliability, Factor 1 showed high values, while Factor 2 reached acceptable levels.

Standardized factor loadings were high and stable across both factors: Factor 1 (items x3–x20) presented values of λ ≈ 0.49–0.77, with several items at or above 0.70; Factor 2 (items x1, x2, x4, and x7) showed loadings of λ ≈ 0.52–0.82. Nevertheless, the average variance extracted (AVE) values for both factors fell slightly below the conventional threshold of 0.50, and the squared inter-factor correlation exceeded both AVE values, indicating limited discriminant validity and suggesting that the factors may represent related dimensions of a broader underlying construct.

The latent correlation between factors in the CFA was high (≈0.79), slightly higher than in the exploratory phase (≈0.59). The difference can be explained by using categorical indicators with thresholds (a more realistic model) and better use of polychoric information in the confirmatory fit. The strong interfactor association indicates that both dimensions reflect a shared underlying vulnerability related to digital overuse, consistent with theoretical models of technostress and self-regulation in technology-intensive work.

### Reliability, convergent validity, and discriminant validity

In terms of internal reliability, an α (ordinal) of 0.93 was obtained for Factor 1 and 0.76 for Factor 2, and an ω of ≈0.91 for Factor 1 and 0.69 for Factor 2.

Values ≥0.70 are considered adequate for research/management use; F2 is borderline due to the brevity of the subscale (four items), which is to be expected (reliability increases with the number of items). Operationally, both factors are sufficiently reliable; if F2 needs to be reinforced, it is suggested that 1–2 additional items consistent with its content be incorporated. From an occupational-psychology perspective, the reliability levels indicate that the instrument can be used for screening and monitoring digital-behavioral risks in frontline emergency personnel.

Regarding convergent validity, factor 1 yielded an AVE of ≈0.47 and factor 2 an AVE of 0.45, both marginally below the conventional 0.50 threshold. These values indicate that, on average, each factor accounts for roughly ~45%−47% of the variance in its items. It is worth noting that AVE tends to be conservative when estimated from items rated on 4–5 point ordinal scales; in this context, the strong loading pattern and high composite reliability (CR) partially offset this limitation. Should stricter psychometric standards be required, one viable course of action would be to refine or replace the items with the lowest factorial loadings. With respect to discriminant validity, the squared inter-factor correlation exceeded both AVE values, suggesting limited differentiation between the two factors and indicating that they may represent related dimensions of a broader underlying construct. Finally, CR values were ≈0.93 for factor 1 and 0.76 for factor 2, reflecting excellent and adequate internal consistency, respectively.

Using the Fornell–Larcker criterion with an *r* ≈ 0.79, we obtain an *r*^2^ of ≈0.62, which is greater than each AVE (0.45–0.47). In other words, the criterion is not met, as the two factors overlap significantly. This overlap is theoretically coherent in safety-critical work, where digital engagement patterns often co-occur and may jointly influence attentional functioning, fatigue, and decision-making.

The practical implication of this is that the two dimensions are distinguishable, since they are structurally separate and the fit of the two-factor model is very good, but they are highly correlated; in practice, they could behave as subscales of a larger common construct. This opens three equally valid options, depending on the objective:

1. Report by subscales (F1 and F2) and a total score (higher-order factor), useful when an overall index is desired.

2. Second-order or bifactor model: confirm that both subscales load on a general dimension; this usually improves interpretability when the correlation is high.

3. If the intervention or analysis requires maximum conceptual separation, consider reviewing the content of the items closest to the boundary between factors to increase their specificity.

The HTMT estimate could not be completed in the session due to an object coercion error; given *r* ≈ 0.79, it is plausible that HTMT > 0.85–0.90, which would reinforce the same conclusion.

### External validity and sample size

The analysis of external validity (criterion and known groups) was not included in this phase, considering external criteria (e.g., objective measures of postural load, cycle times, or clinical indicators) or comparisons by known groups (e.g., jobs with different ergonomic exposure). Future research should correlate scores with ergonomic indicators (RULA/REBA, exposure to vibration/noise, micro-breaks, etc.) and organizational/health outcomes (musculoskeletal discomfort, absenteeism, and productivity). Finding associations in the expected direction will provide additional criterion validity. Such extensions are especially relevant for understanding how digital overuse interacts with cognitive load, fatigue, and operational performance in emergency response work.

With >200 observations for 20 items and typical loadings ≥0.60, the statistical power for the CFA (RMSEA close to 0.03) is adequate, and the confidence intervals for the parameters are narrow.

## Discussion

The present findings should be interpreted against a broader, rapidly evolving landscape in which digital technologies are simultaneously indispensable operational tools and potential sources of maladaptive use. The literature documents how persuasive design, constant connectivity, and reward-based interaction loops can foster compulsive patterns of engagement—what has been variously framed as technoaddiction or problematic Internet/smartphone use (Young, 1998; Kuss and Griffiths, 2011; Alter, 2017; Brand et al., 2019; Lupo et al., 2023). At the same time, organizational research highlights those work-related drivers of overuse (off-hours smartphone demands, blurred boundaries, productivity pressures) are pervasive and consequential for employee well-being and performance (Blake et al., 2024; Lam and Harcourt, 2024; Mouakket and Aboelmaged, 2023). These dynamics illustrate how digital overuse has become a central psychosocial risk in contemporary work, particularly in occupations where attentional functioning and rapid decision-making are critical for safety.

Applying these insights to safety-critical occupations exposes both conceptual and practical tensions. Firefighters rely on mobile devices, geolocation, and real-time information systems that improve situational awareness and coordination (Weidinger, 2022; Weidinger et al., 2023; Fischer-Preßler et al., 2024), yet the same technologies can increase cognitive load, interruptive burden, and susceptibility to maladaptive engagement—risks that are amplified by shift schedules, sleepiness, and alarm-driven work rhythms (Billings et al., 2024; Paterson et al., 2022; Phillips et al., 2024). Empirical work on firefighter risk perception and mental health further suggests that technological stressors interact with traditional occupational stressors (Martínez-Fiestas et al., 2020; Lingard et al., 2024), complicating simple attributions of harm to “addiction” alone. This interaction between digital demands and core job stressors reflects the broader transformation of work, in which technology reshapes professional roles, cognitive requirements, and the boundaries of competent performance.

Methodologically, validating instruments such as the Internet Addiction Test (IAT) for firefighters is necessary but insufficient unless measurement is embedded in occupational realities. Generic scales may conflate adaptive, duty-related device use with problematic patterns driven by compulsivity or poor self-regulation; they may also fail to capture context-dependent trade-offs between information utility and distraction (Moon et al., 2018; Montag et al., 2021). Thus, construct refinement (disentangling functional from dysfunctional use), multimodal assessment (self-report, behavioral telemetry, sleep and fatigue indices), and longitudinal designs are essential to establish criterion validity and to map causal pathways linking technology use to safety outcomes (Pontes et al., 2014; Carbonell et al., 2018; Nastjuk et al., 2024). In the context of the future of work, such refinements are central to understanding how technology-intensive environments demand new forms of self-regulation, attentional control, and digital competence–core components of emerging notions of new professionalism in technology-mediated work.

From a practical and policy perspective, organizations must treat digital overuse as an occupational hazard that requires systems-level responses rather than solely individual remediation. Interventions should combine ergonomic and human-systems engineering (redesigning alerts, prioritizing critical communications), clear organizational norms and policies about acceptable use, and training that builds adaptive decision rules under stress (Tarafdar et al., 2019; Lam and Harcourt, 2024). Given the dual role of ICT in disaster response, any restriction must preserve mission-critical channels while mitigating non-essential interruptions—an approach that aligns with calls for context-sensitive digital governance in emergency services (Weidinger et al., 2023; Fischer-Preßler et al., 2024). These organizational strategies reflect a shift toward proactive governance of digital risks, a hallmark of modern work systems where human–technology interaction is central to performance and safety.

Finally, future research should pursue three priorities. First, develop and validate occupation-specific measurement models that separate operationally necessary use from compulsive patterns and that integrate physiological and performance markers (Moon et al., 2018; Montag et al., 2021). Second, test multilevel interventions that combine technological redesign, organizational policy, and individual support, using experimental or quasi-experimental designs to assess effects on safety, cognition, and wellbeing (Blake et al., 2024; Lam and Harcourt, 2024). Third, investigate moderating influences such as shift schedules, community evacuation demands, and cultural norms around connectivity—factors shown to shape both technology adoption and emergency response outcomes (Zikeloglou et al., 2024; Billings et al., 2024; Lingard et al., 2024). Together, these lines of inquiry can clarify how digitalization reshapes professional identity, risk management, and the competencies required for safe and effective performance in technology-intensive work.

In sum, the present study contributes to broader debates on new professionalism and human–technology interaction by demonstrating how maladaptive patterns of ICT engagement can be identified, monitored, and mitigated in professions where lapses in attention or judgment carry substantial risk. This aligns with contemporary discussions on how technology-intensive work systems reshape professional competencies, risk-management practices, and the very notion of professionalism in the future of work (Lam and Harcourt, 2024; Obasi and Benson, 2025; Fischer-Preßler et al., 2024).

## Conclusions

All participants provided written informed consent in accordance with the Declaration of Helsinki, and the psychometric evidence supports the instrument's suitability for use in safety-critical, technology-intensive occupational settings. Exploratory factor analysis, the heat-map of loadings and the factorial plane consistently delineated two coherent dimensions (F1: items x3–x20; F2: items x1, x2, x4, x7), and confirmatory factor analysis returned excellent fit indices (CFI = 0.997; TLI = 0.997; RMSEA = 0.025; SRMR = 0.063), confirming the stability of a bifactorial structure. Reliability was very good for the primary dimension (F1: α = 0.90; ω = 0.91) and acceptable for the secondary dimension (F2: α = 0.70; ω = 0.69), suggesting that F2 would benefit from additional supporting items to raise internal consistency to the level of F1.

Convergent validity reached acceptable levels (AVE ≈ 0.45–0.47; high composite reliability), while discriminant validity did not fully meet the Fornell–Larcker criterion because of a strong interfactor correlation (*r*^2^ = 0.62). Practically, these results indicate that the scale captures a dominant, coherent construct of problematic technology engagement together with a narrower secondary facet; therefore, reporting both subscale scores and a total score is advisable, and researchers should consider testing a second-order model in advanced applications to reflect the hierarchical relation between the general tendency and its specific manifestations.

From an applied perspective, these findings underscore the relevance of monitoring digital-behavioral risks in occupations where attentional functioning, rapid decision-making, and situational awareness are essential for safety. The validated IAT offers a practical tool for identifying maladaptive patterns of ICT engagement in forest firefighters, contributing to organizational efforts to manage digital demands, prevent cognitive overload, and support healthier human–technology interaction in emergency response work.

The strong association between factors and the modest AVE values highlight the need to complement self-report measures with objective ergonomic, cognitive, and operational indicators—such as alert-handling metrics, fatigue indices, or performance under stress—to strengthen ecological and predictive validity. Integrating psychometric assessment with organizational data aligns with contemporary approaches to digital-risk governance and with emerging models of new professionalism, which emphasize self-regulation, attentional control, and adaptive technology use as core competencies in the future of work.

For future validation and implementation, we recommend (1) augmenting F2 with targeted items to improve reliability for that facet; (2) conducting external validation studies that link scale scores to objective ergonomic measures and operational outcomes (e.g., cognitive performance under heat/stress, shift-related sleepiness, and evacuation decision metrics); and (3) exploring higher-order or bifactor models when the goal is to disentangle a general techno-engagement factor from specific behavioral manifestations. Such efforts will help clarify how digitalization reshapes professional roles, risk-management practices, and the competencies required for safe and effective performance in technology-intensive work environments.

Overall, this study contributes to broader debates on new professionalism and human–technology interaction by demonstrating how problematic ICT engagement can be measured, interpreted, and addressed in safety-critical occupations. The validated IAT provides a foundation for developing evidence-based interventions and organizational policies aimed at promoting safer, healthier, and more sustainable digital work environments in frontline emergency services. This validation clarifies how digitalization reshapes professional practice, human–technology interaction, and the competencies required for safe performance in technology-intensive work environments.

## Data Availability

The original contributions presented in the study are included in the article/Supplementary material, further inquiries can be directed to the corresponding authors.

## References

[B1] Abdul-Hussein A. H. Hussein U. A.-R. Radi U. K. Mahmood H. S. (2023). Prevalence of Internet Addiction Disorder among Iraqi medical populations: a cross-sectional study. Latin Am. J. Pharm. 42, 285–289.

[B2] Alter A. (2017). Irresistible: The Rise of Addictive Technology and the Business of Keeping Us Hooked. New York, NY: Penguin Press.

[B3] Autor D. Mindell D. A. Reynolds E. (2020). The Work of the Future: Building Better Jobs in an Age of Intelligent Machines. Cambridge, MA: MIT Press.

[B4] Aydin E. F. Alay H. Yilmaz S. Can F. K. (2024). The interplay between problematic Internet use, anxiety, depression and functional impairment in front-line healthcare workers during the COVID-19 pandemic. Psychiatry Invest. 21, 736–745. doi: 10.30773/pi.2023.0022 39089699 PMC11298270

[B5] Bartlett M. S. (1950). Tests of significance in factor analysis. Br. J. Stat. Psychol. 3, 77–85. doi: 10.1111/j.2044-8317.1950.tb00285.x

[B6] Bata H. Pentina I. Tarafdar M. Pullins E. B. (2018). Mobile social networking and salesperson maladaptive dependence behaviors. Comput. Hum. Behav. 81:235–249. doi: 10.1016/j.chb.2017.12.025

[B7] Billings J. M. Jahnke S. A. Haddock C. K. (2024). Daily variation in sleepiness among firefighters while working the 24/48 and 48/96 shift schedules. Saf. Sci. 169:106335. doi: 10.1016/j.ssci.2023.106335 39205677 PMC11350525

[B8] Blake H. Hassard J. Singh,. J Teoh K. (2024). Work-related smartphone use during off-job hours and work-life conflict: a scoping review. PLoS Digit. Health 3:e0000554. doi: 10.1371/journal.pdig.0000554 39078844 PMC11288435

[B9] Brand M. Wegmann E. Stark R. Müller A. Wölfling K. Robbins T. W. . (2019). The interaction of person-affect-cognition-execution (I-PACE) model for addictive behaviors: update, generalization to addictive behaviors beyond internet-use disorders, and specification of the process character of addictive behaviors. Neurosci. Biobehav. Rev. 104, 1–10. doi: 10.1016/j.neubiorev.2019.06.032 31247240

[B10] Brand M. Young K. S. Laier C. Wölfling K. Potenza M. N. (2016). Integrating psychological and neurobiological considerations regarding the development and maintenance of specific Internet-use disorders: an interaction of person-affect-cognition-execution (I-PACE) model. Neurosci. Biobehav. Rev. 71, 252–266. doi: 10.1016/j.neubiorev.2016.08.033 27590829

[B11] Carbonell X. Chamarro A. Oberst U. Rodrigo B. Prades M. (2018). Problematic use of the internet and smartphones in university students: 2006–2017. Int. J. Environ. Res. Public Health 15:475. doi: 10.3390/ijerph15030475 29518050 PMC5877020

[B12] Castells M. (2010). The Rise of the Network Society, 2nd Edn. Oxford: Wiley-Blackwell.

[B13] Chen S.-W. Gau S. S.-F. Pikhart H. Peasey A. Shih-Tse C.hen Tsai M.-C. (2014). Work stress and subsequent risk of Internet addiction among information technology engineers in Taiwan. Cyberpsychol. Behav. Soc. Netw. 17:542–50. doi: 10.1089/cyber.2013.0686 24950412

[B14] Cheng X. Fan Y. Li S. Li X. Jin S. Zhou C. . (2025). Research landscape and trends of internet addiction disorder: a comprehensive bibliometric analysis of publications in the past 20 years. Digit. Health 11, 1–17. doi: 10.1177/20552076251336940 PMC12034966 40297375

[B15] Cimino S. Cerniglia L. (2025). Implicit measures of risky behaviors in adolescence. Adolescents 5:77. doi: 10.3390/adolescents5040077

[B16] Comrey A. L. Lee H. B. (1992). A First Course in Factor Analysis, 2nd Edn. Hillsdale, NJ: Lawrence Erlbaum Associates.

[B17] Davis R. A. (2001). A cognitive–behavioral model of pathological internet use. Comput. Hum. Behav. 17, 187–195. doi: 10.1016/S0747-5632(00)00041-8

[B18] Drazich B. F. Nyikadzino Y. Gleason K. T. (2021). A program to improve digital access and literacy among community stakeholders: cohort study. JMIR Form. Res. 5:e30605. doi: 10.2196/30605 34757316 PMC8663502

[B19] Elhai J. D. Yang H. McKay D. Asmundson G. J. G. (2020). COVID-19 anxiety symptoms associated with problematic smartphone use severity in Chinese adults. J. Affect. Disord. 274, 576–582. doi: 10.1016/j.jad.2020.05.080 32663990 PMC7251360

[B20] Elhai J. D. Yang H. Montag C. (2019). Cognitive- and emotion-related dysfunctional coping processes: transdiagnostic mechanisms explaining depression and anxiety's relations with problematic smartphone use. Curr. Addict. Rep. 6, 410–417. doi: 10.1007/s40429-019-00260-4

[B21] Eurofound (2020). Living, Working and COVID-19. Luxembourg: Publications Office of the European Union.

[B22] Fischer-Preßler D. Bonaretti D. Bunker D. (2024). Digital transformation in disaster management: a literature review. J. Strateg. Inf. Syst. 33:101865. doi: 10.1016/j.jsis.2024.101865

[B23] Fornell C. Larcker D. F. (1981). Evaluating structural equation models with unobservable variables and measurement error. J. Mark. Res. 18, 39–50. doi: 10.1177/002224378101800104

[B24] Fukuda M. Chowdhury T. T. Turin Chowdhury T. Tsumura H. Tsuchie R. Kinuta M. . (2023). At-risk internet addiction and related factors among senior high school teachers in Japan based on a nationwide survey. Neuropsychopharmacol. Rep. 43, 553–560. doi: 10.1002/npr2.12350 37465913 PMC10739093

[B25] Gao S. Shao B. (2024). Problematic social media use and employee outcomes: a systematic literature review. Sage Open 14, 1–28. doi: 10.1177/21582440241259158

[B26] García-Iglesias J. J. Bermejo-Ramírez A. M. Goniewicz K. Fernández-Carrasco F. J. Gómez-Salgado C. Camacho-Vega J. C. . (2025). Predictive stressors for the burnout syndrome in firefighters. A systematic review. Saf. Sci. 186:106831. doi: 10.1016/j.ssci.2025.106831

[B27] Gorsuch R. L. (1983). Factor Analysis, 2nd Edn. Hillsdale, NJ: Lawrence Erlbaum Associates.

[B28] Graham J. W. (2009). Missing data analysis: making it work in the real world. Annu. Rev. Psychol. 60, 549–576. doi: 10.1146/annurev.psych.58.110405.085530 18652544

[B29] Griffiths M. D. (2005). A ‘components' model of addiction within a biopsychosocial framework. J. Subst. Use 10, 191–197. doi: 10.1080/14659890500114359

[B30] Grover S. Sahoo S. Bhalla A. Avasthi A. (2019). Problematic internet use and its correlates among resident doctors of a tertiary care hospital of North India: a cross-sectional study. Asian J. Psychiatry 39, 42–47. doi: 10.1016/j.ajp.2018.11.018 30529568

[B31] Henseler J. Ringle C. M. Sarstedt M. (2015). A new criterion for assessing discriminant validity in variance-based structural equation modeling. J. Acad. Mark. Sci. 43, 115–135. doi: 10.1007/s11747-014-0403-8

[B32] Horn J. L. (1965). A rationale and test for the number of factors in factor analysis. Psychometrika 30, 179–185. doi: 10.1007/BF02289447 14306381

[B33] Howard S. J. Hayes N. Mallawaarachchi S. Johnson D. Neilsen-Hewett C. Mackenzie J. . (2025). A meta-analysis of self-regulation and digital recreation from birth to adolescence. Comput. Hum. Behav. 163:108472. doi: 10.1016/j.chb.2024.108472

[B34] Hu L. T. Bentler P. M. (1999). Cutoff criteria for fit indexes in covariance structure analysis: conventional criteria versus new alternatives. Struct. Equ. Modeling 6, 1–55. doi: 10.1080/10705519909540118

[B35] Hu Y. (2025). School engagement as mediator between mobile device usage and social-emotional development for left-behind children in China. Discov. Psychol. 5:196. doi: 10.1007/s44202-025-00507-4

[B36] Huang K. Yang Y. Wang L. Li J. Qu D. Chen R. . (2026). The dual effects of individual and contextual factors on adolescent problematic internet use: machine learning approaches and SHAP explanations. J. Behav. Addict. 15, 274–288. doi: 10.1556/2006.2025.00160 41805694 PMC13132359

[B37] Kaiser H. F. Rice J. (1974). Little Jiffy, Mark IV. Educ. Psychol. Meas. 34, 111–117. doi: 10.1177/001316447403400115

[B38] Kambarian E. Butler P. C. Cohen-Hatton S. R. Honey R. C. (2025). Contrasting safety attitudes, behaviors and practices in US and UK firefighters. Saf. Sci. 189:106884. doi: 10.1016/j.ssci.2025.106884

[B39] Kuss D. J. Griffiths M. D. (2011). Online social networking and addiction: a review of the psychological literature. Int. J. Environ. Res. Public Health 8, 3528–3552. doi: 10.3390/ijerph8093528 22016701 PMC3194102

[B40] Lam H. Harcourt M. (2024). Digital addiction in organizations: challenges and policy implications. Employ. Respons. Rights J. 36, 519–533. doi: 10.1007/s10672-024-09493-6

[B41] Laudon K. C. Laudon J. P. (2020). Management Information Systems: Managing the Digital Firm, 16th Edn. Harlow: Pearson.

[B42] Li C.-H. (2016). Confirmatory factor analysis with ordinal data: comparing robust maximum likelihood and diagonally weighted least squares. Behav. Res. Methods 48, 936–949. doi: 10.3758/s13428-015-0619-7 26174714

[B43] Li G. L. Jin C. F. Zhao B. Wu B. (2022). Smartphone use, technology affordance for healthcare and elders' life satisfaction. Front. Public Health 10:861897. doi: 10.3389/fpubh.2022.861897 35480578 PMC9035850

[B44] Lingard H. Hayes P. Turner M. (2024). Work-related risk factors for mental ill-health among Australian wildland firefighters. Saf. Sci. 178:106619. doi: 10.1016/j.ssci.2024.106619

[B45] Lupo R. Vitale E. Carriero M. C. Calabrò A. Imperiale C. Ercolani M. . (2023). Gambling and internet addiction: a pilot study among a population of Italian healthcare workers. J. Gambl. Stud. 39, 1337–1354. doi: 10.1007/s10899-022-10150-6 35908025 PMC9362545

[B46] MacCallum R. C. Widaman K. F. Zhang S. Hong S. (1999). Sample size in factor analysis. Psychol. Methods 4, 84–99. doi: 10.1037/1082-989X.4.1.84 26822184

[B47] Martínez-Fiestas M. Rodríguez-Garzón I. Delgado-Padial A. (2020). Firefighter perception of risk: a multinational analysis. Saf. Sci. 123:104545. doi: 10.1016/j.ssci.2019.104545

[B48] Mekonnen Y. S. Tessema S. A. Bedane S. D. Ali A. B. (2024). Problematic Internet use among resident physicians at St. Paul's Hospital Millennium Medical College in Addis Ababa, Ethiopia. BMC Psychiatry 24:960. doi: 10.1186/s12888-024-06390-y 39741254 PMC11687078

[B49] Montag C. Wegmann E. Sariyska R. Demetrovics Z. Brand M. (2021). How to overcome taxonomical problems in the study of internet use disorders and what to do with “smartphone addiction”? J. Behav. Addict. 9, 908–914. doi: 10.1556/2006.8.2019.59 31668089 PMC8969715

[B50] Moon S. J. Hwang J. S. Kim J. Y. Shin A. L. Bae S. M. Kim J. W. (2018). Psychometric properties of the internet addiction test: a systematic review and meta-analysis. Cyberpsychol. Behav. Soc. Netw. 21, 473–484. doi: 10.1089/cyber.2018.0154 30110200

[B51] Mouakket S. Aboelmaged M. (2023). Understanding the influence of problematic smartphone use on work productivity in the post-COVID-19 pandemic. Inf. Dev. 42, 313–333. doi: 10.1177/02666669231213940

[B52] Muñoz-Rivas M. J. Fernández L. Gámez-Guadix M. (2010). Analysis of the indicators of pathological internet use in Spanish university students. Span. J. Psychol. 13, 697–707. doi: 10.1017/S1138741600002365 20977019

[B53] Nastjuk I. Trang S. Grummeck-Braamt J. V. Adam M. T. P. Tarafdar M. (2024). Integrating and synthesising technostress research: a meta-analysis on technostress creators, outcomes, and IS usage contexts. Eur. J. Inf. Syst. 33, 361–382. doi: 10.1080/0960085X.2022.2154712

[B54] Nunnally J. C. Bernstein I. H. (1994). Psychometric Theory, 3rd Edn. New York, NY: McGraw-Hill.

[B55] Obasi I. C. Benson C. (2025). The impact of digitalization and information and communication technology on the nature and organization of work and the emerging challenges for occupational safety and health. Int. J. Environ. Res. Public Health 22:362. doi: 10.3390/ijerph22030362 40238361 PMC11942091

[B56] Pagano M. Bacaro V. Crocetti E. (2023). “Using digital media or sleeping … that is the question”. A meta-analysis on digital media use and unhealthy sleep in adolescence. Comput. Hum. Behav. 146:107813. doi: 10.1016/j.chb.2023.107813

[B57] Park S. (2026). Wealth, digital overuse, and the changing landscape of digital inequality. Comput. Hum. Behav. 174:108805. doi: 10.1016/j.chb.2025.108805

[B58] Paterson J. L. Aisbett B. Kovac K. Ferguson S. A. (2022). Informal management of health and safety risks associated with alarm response by Australian firefighters. Ergonomics 65, 233–241. doi: 10.1080/00140139.2021.1967460 34429036

[B59] Phillips J. G. Chow Y. W. Ogeil R. P. (2024). Decisional style, sleepiness, and online responsiveness. Ergonomics 67, 1177–1189. doi: 10.1080/00140139.2023.2288808 38006288

[B60] Pontes H. M. (2017). Investigating the differential effects of social networking site addiction and internet gaming disorder on psychological health. J. Behav. Addict. 7, 601–610. doi: 10.1556/2006.6.2017.075 29130329 PMC6034963

[B61] Pontes H. M. Patrao I. M. Griffiths M. D. (2014). Portuguese validation of the internet addiction test: an empirical study. J. Behav. Addict. 3, 107–114. doi: 10.1556/JBA.3.2014.2.4 25215221 PMC4117285

[B62] Qi H. Song D. Wang J. Li J. Qu G. Chen X. . (2025). Dysfunctional reward processing amplifies stress-related smartphone overuse: evidence from ERPs and ecological momentary assessment. J. Behav. Addict. 14, 1429–1443. doi: 10.1556/2006.2025.00066 40875484 PMC12486276

[B63] R Core Team Z. Z. (2025). R: A Language and Environment for Statistical Computing (Version 4.4.3). Vienna: R Foundation for Statistical Computing. Available online at: https://www.R-project.org/ (Accessed January 15, 2026).

[B64] Raykov T. (1997). Estimation of composite reliability for congeneric measures. Appl. Psychol. Meas. 21, 173–184. doi: 10.1177/01466216970212006

[B65] Revelle W. (2024). Psych: Procedures for Psychological, Psychometric, and Personality Research. Evanston, IL: Northwestern University.

[B66] Rhemtulla M. Brosseau-Liard P. É. Savalei V. (2012). When can categorical variables be treated as continuous? A comparison of robust continuous and categorical SEM estimation methods under suboptimal conditions. Psychol. Methods 17, 354–373. doi: 10.1037/a0029315 22799625

[B67] Rönkkö M. Cho E. (2022). An updated guideline for assessing discriminant validity. Organ. Res. Methods 25, 6–14. doi: 10.1177/1094428120968614

[B68] Rosseel Y. (2012). lavaan: an R package for structural equation modeling. J. Stat. Softw. 48, 1–36. doi: 10.18637/jss.v048.i02

[B69] Servidio R. Koronczai B. Griffiths M,. D Demetrovics Z. (2022). Problematic smartphone use and problematic social media use: the predictive role of self-construal and the mediating effect of fear missing out. Front. Public Health 10:814468. doi: 10.3389/fpubh.2022.814468 35284373 PMC8904752

[B70] Shi D. Maydeu-Olivares A. Rosseel Y. (2020). Assessing fit in ordinal factor analysis models: SRMR vs. RMSEA. Struct. Equ. Modeling 27, 1–15. doi: 10.1080/10705511.2019.1611434

[B71] Sun H.-L. Chen P. Zhang Q. Sia T. L. Li Y.-Z. Zhu H.-Y. . (2024). Prevalence and network analysis of internet addiction, depression and their associations with sleep quality among commercial airline pilots: a national survey in China. J. Affect. Disord. 356, 597–603. doi: 10.1016/j.jad.2024.03.022 38484881

[B72] Tarafdar M. Cooper C. L. Stich J. F. (2019). The technostress trifecta: techno eustress, techno distress and design. Inf. Syst. J. 29, 6–42. doi: 10.1111/isj.12169

[B73] Troyanskaya O. Cantor M. Sherlock G. Brown P. Hastie T. Tibshirani R. . (2001). Missing value estimation methods for DNA microarrays. Bioinformatics 17, 520–525. doi: 10.1093/bioinformatics/17.6.520 11395428

[B74] UNESCO Z. Z. (2009). Towards Information Literacy Indicators. Paris: United Nations Educational, Scientific and Cultural Organization.

[B75] van Buuren S. Groothuis-Oudshoorn K. (2011). Mice: multivariate imputation by chained equations in R. J. Stat. Softw. 45, 1–67. doi: 10.18637/jss.v045.i03

[B76] Vanea M. O. (2011). Intensive/excessive use of internet and risks of internet addiction among specialized workers—gender and online activities differences. Proc. Soc. Behav. Sci. 30, 757–764. doi: 10.1016/j.sbspro.2011.10.148

[B77] Weidinger J. (2022). What is known and what remains unexplored: a review of the firefighter information technologies literature. Int. J. Disaster Risk Reduct. 78:103115. doi: 10.1016/j.ijdrr.2022.103115

[B78] Weidinger J. Schlauderer S. Overhage S. (2023). Information technology to the rescue? Explaining the acceptance of emergency response information systems by firefighters. IEEE Trans. Eng. Manag. 70, 14–28. doi: 10.1109/TEM.2020.3044720

[B79] Widyanto L. McMurran M. (2004). The psychometric properties of the internet addiction test. Cyberpsychol. Behav. 7, 443–450. doi: 10.1089/cpb.2004.7.443 15331031

[B80] Young K. S. (1998). Internet addiction: the emergence of a new clinical disorder. Cyberpsychol. Behav. 1, 237–244. doi: 10.1089/cpb.1998.1.237

[B81] Zikeloglou I. Lekkas E. Lozios S. Stavropoulou M. (2024). Community's evacuation planning and response for the 2021–2022 fire seasons in Greece. Saf. Sci. 172:106434. doi: 10.1016/j.ssci.2024.106434

[B82] Zinbarg R. E. Revelle W. Yovel I. Li W. (2005). Cronbach's α, Revelle's β, and McDonald's ωH: their relations with each other and two alternative conceptualizations of reliability. Psychometrika 70, 123–133. doi: 10.1007/s11336-003-0974-7

